# Ochratoxin A Induces Steatosis via PPARγ-CD36 Axis

**DOI:** 10.3390/toxins13110802

**Published:** 2021-11-13

**Authors:** Qian-Wen Zheng, Xu-Fen Ding, Hui-Jun Cao, Qian-Zhi Ni, Bing Zhu, Ning Ma, Feng-Kun Zhang, Yi-Kang Wang, Sheng Xu, Tian-Wei Chen, Ji Xia, Xiao-Song Qiu, Dian-Zhen Yu, Dong Xie, Jing-Jing Li

**Affiliations:** 1 CAS Key Laboratory of Nutrition, Metabolism and Food Safety, Shanghai Institute of Nutrition and Health, University of Chinese Academy of Sciences, Chinese Academy of Sciences, Shanghai 200031, China; zhengqw@shanghaitech.edu.cn (Q.-W.Z.); xfding@sibs.ac.cn (X.-F.D.); caohuijun2017@sibs.ac.cn (H.-J.C.); qzni@sibs.ac.cn (Q.-Z.N.); zhubing2018@sibs.ac.cn (B.Z.); maning@sibs.ac.cn (N.M.); zhangfengkun2017@sibs.ac.cn (F.-K.Z.); wangyikang2017@sibs.ac.cn (Y.-K.W.); xusheng2018@sibs.ac.cn (S.X.); chentianwei@sibs.ac.cn (T.-W.C.); xiaji2019@sibs.ac.cn (J.X.); qiuxs@shanghaitech.edu.cn (X.-S.Q.); dzyu@sibs.ac.cn (D.-Z.Y.); 2 School of Life Science and Technology, ShanghaiTech University, Shanghai 201210, China; 3 NHC Key Laboratory of Food Safety Risk Assessment, China National Center for Food Safety Risk Assessment, Beijing 100022, China

**Keywords:** fatty liver disease, lipid metabolism, OTA, PPAR

## Abstract

Ochratoxin A(OTA) is considered to be one of the most important contaminants of food and feed worldwide. The liver is one of key target organs for OTA to exert its toxic effects. Due to current lifestyle and diet, nonalcoholic fatty liver disease (NAFLD) has been the most common liver disease. To examine the potential effect of OTA on hepatic lipid metabolism and NAFLD, C57BL/6 male mice received 1 mg/kg OTA by gavage daily. Compared with controls, OTA increased lipid deposition and TG accumulation in mouse livers. In vitro OTA treatment also promoted lipid droplets accumulation in primary hepatocytes and HepG2 cells. Mechanistically, OTA prevented PPARγ degradation by reducing the interaction between PPARγ and its E3 ligase SIAH2, which led to activation of PPARγ signaling pathway. Furthermore, downregulation or inhibition of CD36, a known of PPARγ, alleviated OTA-induced lipid droplets deposition and TG accumulation. Therefore, OTA induces hepatic steatosis via PPARγ-CD36 axis, suggesting that OTA has an impact on liver lipid metabolism and may contribute to the development of metabolic diseases.

## 1. Introduction

Ochratoxin A (OTA) is produced by several species of Aspergillus and Penicillium [1], and is one of the most common mycotoxin contaminant in food. It has been identified in various crops, including cereals and cereal products, coffee beans, peanuts, dried fruits, spices, legumes, wine and beer [2]. OTA has long been studied as a nephrotoxin, immunotoxin, teratogen and carcinogen in humans as well as other animal species [3,4,5,6], and is regarded to be a nonnegligible risk of human health because of its widespread occurrence. This mycotoxin is metabolized and accumulated mainly in the liver and kidney [7], which are the major target organs for OTA [8].

Liver is a vital metabolic organ in the maintenance of whole-body homeostasis. Because liver is responsible for metabolism, distribution and excretion of exogenous chemicals, it is threatened by significant concentrations of chemicals, and chemical- or drug-induced liver injury (hepatotoxicity). Furthermore, it is recently suggested that nonalcoholic fatty liver disease (NAFLD), or steatosis, is the most prevalent pathology associated with toxicant exposure [9]. In particular, OTA affects hepatocytes via multiple pathways, including oxidative stress [10,11], inflammation [12], apoptosis [13,14,15] and genotoxic effect [16,17], It is reported that OTA would increase the expression of genes involved in the synthesis of fatty acid in kidney. In contrast, it significantly inhibited the expression of genes related to fatty acid oxidation [18]. However, the lipotoxicity of OTA in liver remains unknown.

Peroxisome proliferator-activated receptors (PPARs) are nuclear hormone receptors that are activated by fatty acids and their derivatives [19]. There are three PPAR isotypes—α, β/δ and γ, they are well known to serve as important regulatory factors of lipid metabolism. PPARα modulates transcription of specific target genes involved in lipid oxidation, lipid transport, lipoprotein assembly and ketogenesis [20]. PPARβ/δ is most abundant in metabolically active tissues such as skeletal and cardiac muscle, and regulates lipid metabolism, inflammation and oxidative stress responses [21,22]. PPARγ plays a role in regulating adipocyte differentiation and energy storage in mature adipocytes [23]. Thus, PPARs are promising drug targets for the management of NAFLD.

In the present study, we investigated the influence of OTA on hepatic lipid metabolism. We found that OTA increased lipid droplets deposition and TG accumulation in primary hepatocytes and HepG2 cells, and induced steatosis in mice. The mechanistic study revealed that OTA disturbed lipid metabolism in liver cells mainly through PPARγ-CD36 axis. OTA can stabilize PPARγ via preventing its ubiquitination and subsequent degradation. Therefore, our study provides novel insights into the mechanism underlying the disturbance of hepatic lipid metabolism by OTA.

## 2. Results

### 2.1. Effects of OTA on Lipid Accumulation In Vitro

**Figure 1:**
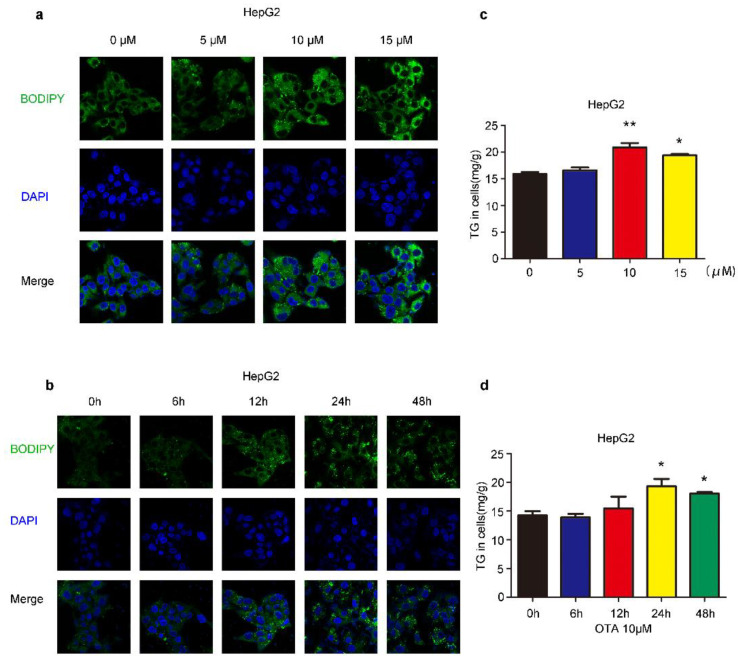
Effects of OTA on lipid accumulation in HepG2 cells. HepG2 cells are treated with OTA at the concentrations of 5, 10 and 15 μM for 24 h, then intracellular lipid droplets are labeled with BODIPY (**a**) and TG contents are determined (**c**). HepG2 cells are treated with OTA at concentrations of 10 μM for 6, 12, 24 and 48 h, then intracellular lipid droplets are labeled with BODIPY (**b**) and TG contents are determined (**d**). Data shown as the mean ± S.E.M. * *p* < 0.05 and ** *p* < 0.01, *n* = 6 biological replicates.

**Figure 2:**
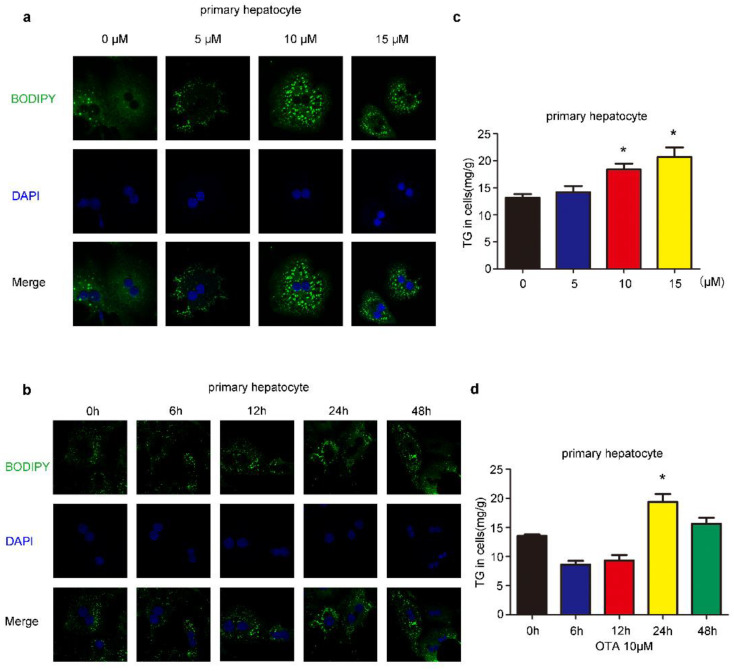
Effects of OTA on lipid accumulation in primary hepatocytes. (**a**) Primary hepatocytes are treated with OTA at the concentrations of 5, 10 and 15 μM for 24 h, then intracellular lipid droplets are labeled with BODIPY (**a**) and TG contents are determined (**c**). Primary hepatocytes are treated with OTA at concentrations of 10 μM for 6, 12, 24 and 48 h, then intracellular lipid droplets are labeled with BODIPY (**b**) and TG contents are determined (**d**). Data shown as the mean ± S.E.M. * *p* < 0.05, *n* = 6 biological replicates.

We first investigated the influences of OTA on liver cells, we treated HepG2 cells by OTA with different doses and exposure times. After treatment, intracellular lipid droplets were labeled with BODIPY to examine lipid accumulation. OTA promoted lipid droplets deposition in a dose-dependent manner, and more lipid droplets were observed in the cells treated with 5 μM and 10 μM OTA (Figure 1a). However, OTA at 15 μM did not further increase intracellular lipid droplets, suggesting that OTA may reach saturation concentration at 10 μM. Moreover, we isolated primary hepatocytes from 8–12 weeks-old mice by liver perfusion. Primary hepatocytes also formed more lipid droplets after OTA treatment in a similar manner (Figure 2a). Based on these observations, we used 10 μM in the following experiments. Additionally, we compared the effects of OTA treatments with different exposure times on HepG2 cells. OTA exerted the most significant effect on lipid droplets formation at 24 h (Figure 1b). A similar manner was found in primary hepatocytes (Figure 2b). We also tested the cellular triacylglycerol (TG) contents after OTA treatment. Consistent with the change in lipid droplets, the cellular TG contents were markedly increased in both HepG2 and primary hepatocytes treated with 10 μM OTA at 24 h when compared with cells under other treatment conditions (Figure 1c,d and Figure 2c,d).

### 2.2. OTA Promotes Development of Hepatic Steatosis in Mice

**Figure 3:**
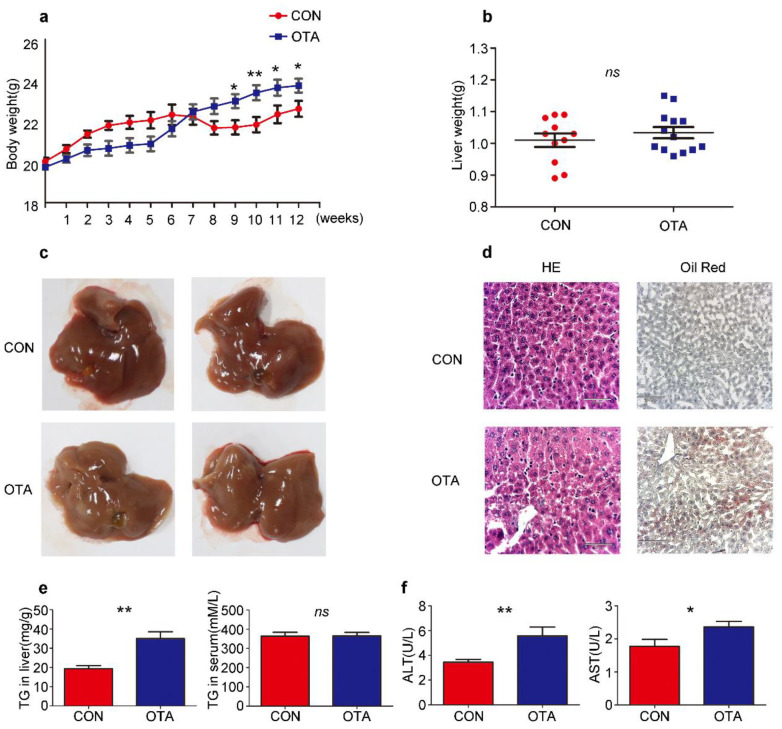
OTA induces simple steatosis in vivo. Mice are treated with OTA or NaHCO3 for 12 weeks (n = 12 in each group). Effects of OTA on mouse: body weight (**a**), liver weight (**b**), representative images of livers (**c**), H&E and Oil Red O staining of liver sections of control and OTA-treated mice (**d**), liver TG contents and serum TG levels (**e**), serum ALT levels and AST levels (**f**). Data shown as the mean ± S.E.M. * *p* < 0.05 and ** *p* < 0.01, *ns* means no significant difference.

To confirm the effects of OTA in vivo, six-week-old C57BL/6 mice were daily administered with OTA at 1 mg/kg.bw in 0.1 M NaHCO_3_ (100 μL) for successively 12 weeks (OTA group). Control mice (Control group) received 0.1 M NaHCO_3_ by gavage similarly. Although OTA mice exhibited slower weight gain than control mice at the early stage, they had a more rapid rise in the body weight after 9-week feeding, suggesting that the long-term effect of OTA exposure on metabolism (Figure 3a). Although liver weights were comparable between control and OTA group (Figure 3b), the livers in OTA group showed a slightly white appearance (Figure 3c). H&E staining of the livers in OTA group showed symptom of fatty liver at early stage (Figure 3d). Furthermore, Oil Red O staining of liver sections revealed significant lipid accumulation in the hepatocytes of mice in OTA group (Figure 3d). Besides, the hepatic TG content was significantly increased in OTA group (Figure 3e), whereas the serum levels of TG in control and OTA groups were comparable (Figure 3e). These findings suggested that OTA treatment led to abnormal lipid metabolism in mice livers. In addition, serum ALT levels and AST levels were increased in OTA group (Figure 3f), indicating liver damage. The above data suggested that 12-week exposure to OTA induced simple steatosis in vivo.

### 2.3. OTA Induces Hepatic Steatosis through PPARγ Signaling

**Figure 4:**
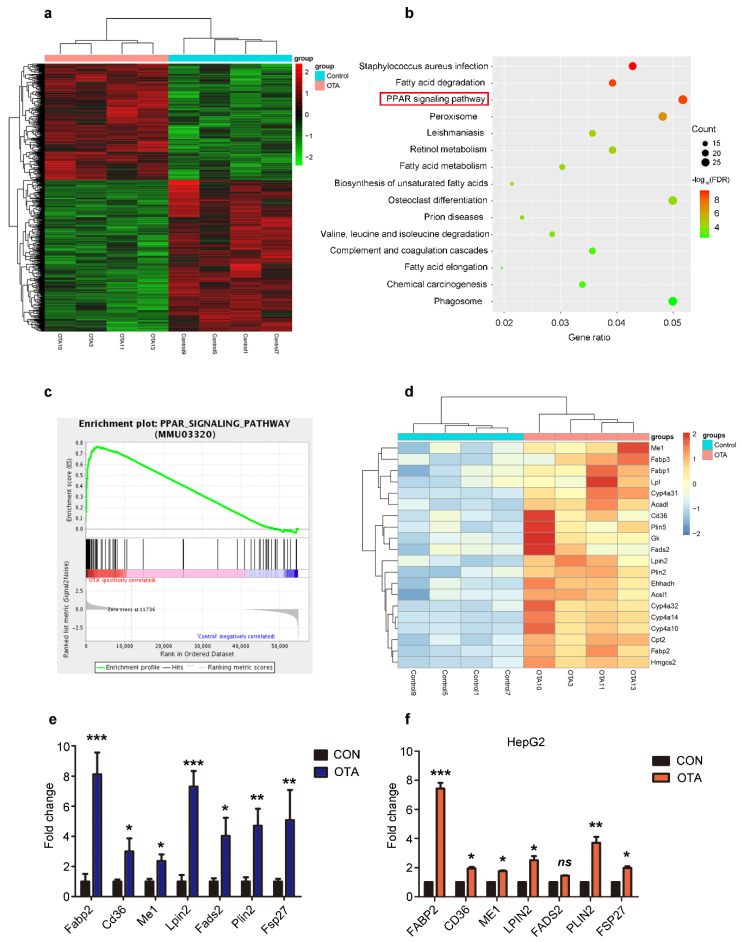
Reprogramming of hepatic gene expression after OTA treatment. (**a**) Heatmap for clustering analysis of genes in the livers of control and OTA-treated mice. (**b**) KEGG analysis showing top 15 enriched pathways in OTA-treated livers with *p* < 0.05. (**c**) Results of GSEA showed PPAR signaling pathway were differentially enriched upon OTA treatment. (**d**) Heatmaps of hepatic RNA-seq raw gene counts for PPAR signaling pathway. (**e**) Examination of top upregulated genes associated with lipid metabolism in livers of control and OTA-treated mice (*n* = 12 in each group) by qPCR. (**f**) Examination of top upregulated genes associated with lipid metabolism in HepG2 cells by qPCR (*n* = 6 biological replicates). Data shown as the mean ± S.E.M. * *p* < 0.05, ** *p* < 0.01 and *** *p* < 0.001.

To identify the mechanisms underlying the effects of OTA on hepatic steatosis, we performed next-generation transcriptome sequencing of the liver samples from mice in control and OTA groups. As expected, a differential gene expression pattern was obtained (Figure 4a). KEGG enrichment analysis showed that mRNA levels of genes in PPAR signaling pathway predominantly altered after OTA treatment (Figure 4b). GSEA enrichment analysis further confirmed the increase in the mRNA level of PPAR signaling pathway-associated genes in OTA group (Figure 4c). We were particularly interested in the enrichment of the lipid droplet-associated genes, which were enriched in PPARγ signaling (Figure 4d). Among them, *Cd36* and *Fabp2* are responsible for lipid uptake; *Me1*, *Lpin2* and *Fads2* are related to lipogenesis; and *Plin2* and *Fsp27* are involved in lipid droplets assembly. We verified their expression by qRT-PCR, and the results confirmed that the mRNA levels of above genes were dramatically increased in the livers of OTA-treated mice (Figure 4e). Interestingly, OTA also upregulated these genes expression in HepG2 cells, except *FADS2* (Figure 4f). Based on these data, we speculated that OTA influenced hepatic lipid metabolism by activation of PPARγ signaling.

**Figure 5:**
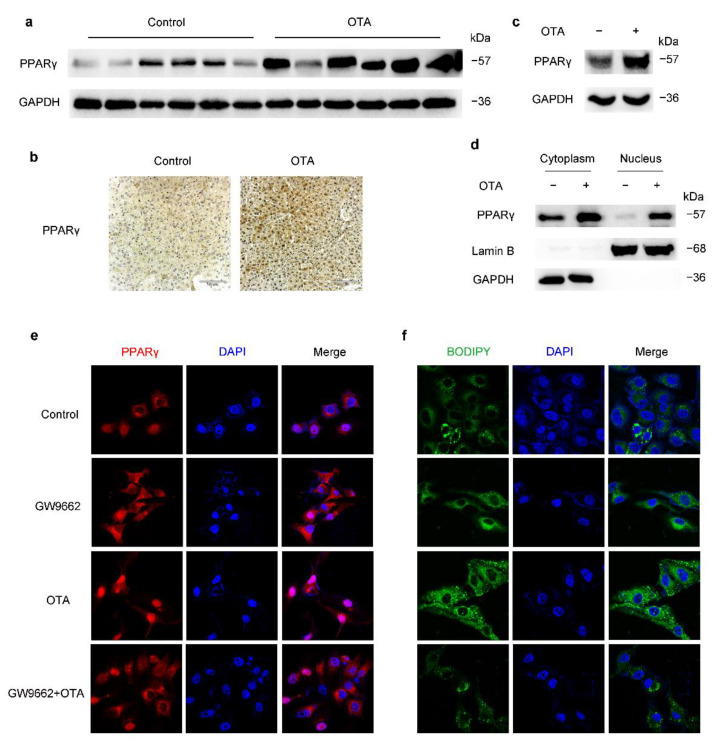
OTA induces hepatic steatosis through upregulation and activation of PPARγ. (**a**) Western blot analysis of PPARγ expression in livers of control and OTA-treated mice. (**b**) Immunohistochemical staining of PPARγ in liver tissues of control and OTA-treated mice. (**c**) PPARγ protein expression in control and OTA-treated HepG2 cells. (**d**) Western blot analysis showing effect of OTA on PPARγ expression in cytosolic and nuclear fractions of HepG2 cells. (**e**) Expression and localization of PPARγ under indicated treatment is examined by immunofluorescence. Nuclei were stained with DAPI, and (**f**) intracellular lipid droplets are labeled with BODIPY.

We found that the protein levels of PPARγ in the livers of OTA-treated mice were markedly increased compared with those of control mice (Figure 5a). Immunohistochemical analysis confirmed strong PPARγ nuclear staining in liver tissues of OTA-treated mice (Figure 5b), and PPARγ-positive cells were much more in the livers of OTA-treated mice than those of control mice (Figure 5b). Besides, PPARγ expression also increased in HepG2 cells after OTA treatment (Figure 5c), accompanied by PPARγ activation, which was indicated by nuclear accumulation of PPARγ protein upon OTA treatment (Figure 5d). Therefore, OTA not only increased PPARγ expression but also promoted its activation.

To confirm whether PPARγ played an important role in OTA-induced steatosis, we treated HepG2 cells with GW9662, a potent antagonist of PPARγ. As expected, GW9662 decreased nuclear PPARγ, and suppressed its activation induced by OTA (Figure 5e). Meanwhile, GW9662 prevented lipid accumulation upon OTA treatment (Figure 5f), which confirmed that PPARγ was involved in the prosteatotic role of OTA. Therefore, increased protein expression and activation of PPARγ were responsible for OTA-induced steatosis.

### 2.4. OTA Regulates PPARγ Protein Stability via Ubiquitin E3 Ligase SIAH2

**Figure 6:**
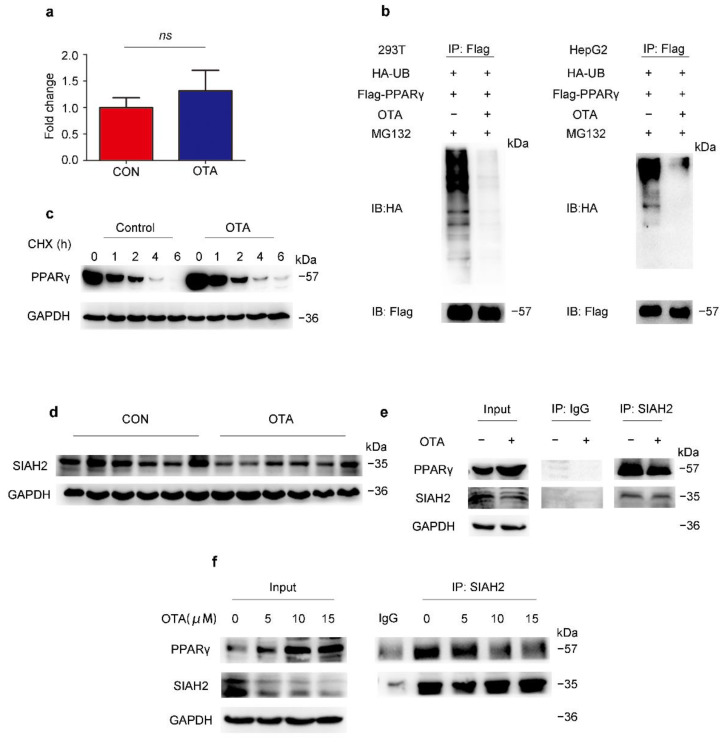
OTA regulates PPARγ by post-translational modification. (**a**) PPARγ mRNA expression in livers of control and OTA-treated mice (*n* = 12 in each group). (**b**) Influence of OTA on the ubiquitination of PPARγ is examined in HEK293T and HepG2 cells. (**c**) PPARγ expression after OTA and cycloheximide (CHX) treatment. (**d**) Western blot analysis of SIAH2 expression in livers of control and OTA-treated mice. (**e**) Endogenous interaction between PPARγ and SIAH2 is examined by immunoprecipitation with SIAH2 antibody in control and OTA-treated HepG2 cells. (**f**) HepG2 cells are treated with OTA at the concentrations of 5, 10 and 15 μM for 24 h, and endogenous interaction between PPARγ and SIAH2 is examined by immunoprecipitation with SIAH2 antibody. Data shown as the mean ± S.E.M. *ns* means no significant difference.

Although the protein level of PPARγ was upregulated by OTA, the PPARγ mRNA level of OTA-treated mice was not statistically significant different from control mice (Figure 6a). Therefore, we speculated that OTA may regulate PPARγ at post-translational level. Ubiquitination is an important modification of PPARγ, thus we examined the effect of OTA on PPARγ ubiquitination. As shown in Figure 6b, we found that OTA dramatically reduced the ubiquitination of PPARγ in 293T and in HepG2 cells (Figure 6b). In the absence of de novo protein synthesis with cycloheximide treatment, the protein level of PPARγ level in OTA-treated HepG2 cells declined more slowly compared with control cells (Figure 6c). To explore the underlying mechanism, we examined the protein level of SIAH2, a previously reported E3 ligase for PPARγ, in the livers of control and OTA group [24], and found that OTA decreased the expression levels of SIAH2 (Figure 6d). Then we examined whether OTA also affected binding of SIAH2 to PPARγ. As shown in Figure 6e, endogenous PPARγ was co-precipitated with SIAH2, however, this interaction was disturbed by the OTA treatment (Figure 6e). Furthermore, we confirmed the SIAH2 expression, as well as SIAH2-PPARγ interaction was suppressed by OTA in a dose-dependent manner, associated with gradually increased PPARγ expression (Figure 6f). We noted that treatment with OTA at 15 μM did not further enhance the inhibitory effect on SIAH2-PPARγ interaction, and this was consistent with the observation that OTA at 15 μM could not further increase intracellular lipid droplets. Thus, these results suggested that OTA upregulated PPARγ expression by interfering with SIAH2-mediated ubiquitination of PPARγ.

### 2.5. OTA Induces Hepatic Steatosis in a CD36-Dependent Manner

**Figure 7:**
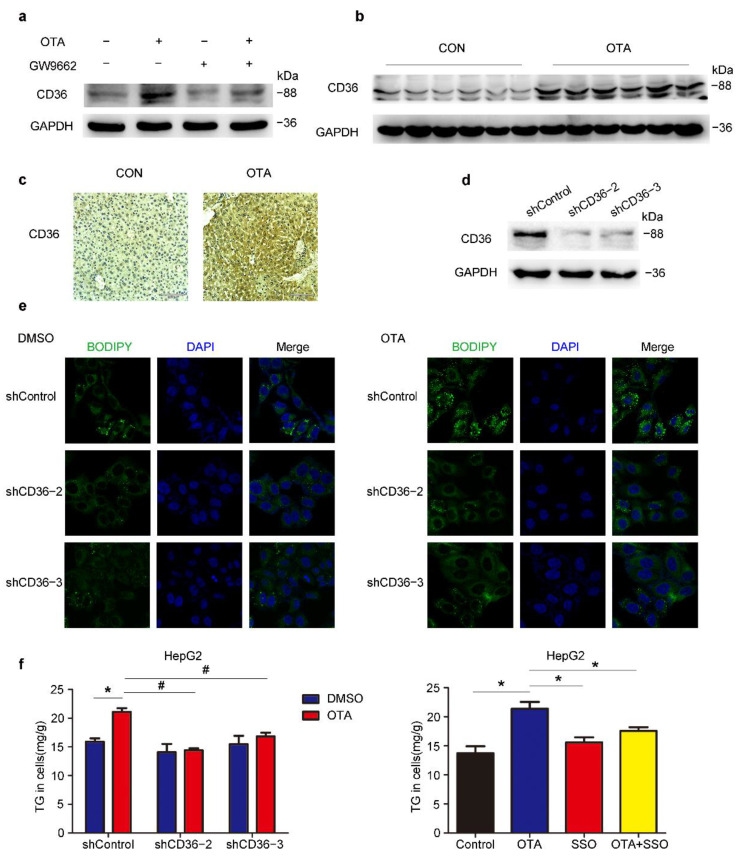
OTA-induced hepatic steatosis is CD36-dependent. (**a**) Western blot analysis of CD36 expression in HepG2 cells under indicated treatment. (**b**) Western blot analysis of CD36 expression in livers of control and OTA-treated mice. (**c**) Immunohistochemical staining of CD36 in liver tissues of control and OTA-treated mice. (**d**) Knockdown efficiency of CD36 in HepG2 cells. (**e**) BODIPY staining of lipid droplets in control and CD36-knockdown HepG2 cells treated with DMSO and OTA. (**f**) Left, TG contents in control and CD36-knockdown HepG2 cells treated with DMSO and OTA. Right, TG contents in HepG2 cells under indicated treatment (*n* = 6 biological replicates). Data shown as the mean ± S.E.M. * *p* <0.05 vs. shControl DMSO treatment; and # *p* < 0.05 vs. shControl OTA treatment.

To further clarify the mechanism underlying the regulation of PPARγ signaling and lipid metabolism by OTA, we checked the expression of PPARγ target genes upon OTA and GW9662 treatment, and found that only FABP2 and CD36, which were involved in lipid uptake were upregulated by OTA alone and reduced by combined treatment of GW9662 and OTA (Figure S1). Similarly, the protein expression of CD36 and FABP2 showed consistent changes (Figure 7a and Figure S2A). The upregulation of CD36 at the protein level was further confirmed by western blots and IHC staining in liver tissues (Figure 7b,c). Then we constructed three *CD36*-specific shRNAs to silence the endogenous CD36 expression in HepG2 cells. The knockdown efficiency of sh*CD36*-2 and sh*CD36*-3 was validated by western blot (Figure 7d). As shown by BODIPY staining, we found that CD36 knockdown slightly reduced lipid droplets accumulation in the absence of OTA, however, CD36 silencing significantly decreased lipid droplets upon OTA treatment in HepG2 cells (Figure 7e). Consistent with our previous observation, OTA increased TG accumulation in shControl cells (Figure 7f), while knockdown of CD36 attenuated the effect of OTA on TG accumulation when compared with shControl cells (Figure 7f). Moreover, pre-incubation of a CD36 inhibitor, sulfosuccinimidyl oleate (SSO), also reduced OTA-induced cellular TG accumulation, further confirming that OTA-induced hepatic steatosis was dependent on CD36. Similarly, we infected HepG2 cells with lentivirus-sh*FABP2* to specifically knockdown FABP2 expression (Figure S2B). We also found that sh*FABP2* inhibited lipid droplets accumulation in HepG2 cells with or without OTA treatment (Figure S2C). However, FABP2 silencing had no significant effect on the TG contents upon OTA treatment (Figure S2D). Collectively, these findings suggested that CD36 and FABP2 mediated lipid metabolic response to OTA. Considering a functional compensation for FABP2, OTA may promote lipid accumulation mainly through CD36 in the liver.

## 3. Discussion

OTA is considered to be one of the most important contaminants of global food and crops. Ambient temperature, humidity, food storage and transportation may promote fungal growth leading to increased occurrence of OTA in various crops [25]. OTA has been detected in human blood and serum in Canada, Sweden, West Germany and Yugoslavia [26], suggesting the high incidence of OTA exposure in human. Therefore, there is a need to investigate the toxic effects of OTA for prevention.

Previous studies reported that inhibition on protein synthesis and energy generation, induction of oxidative stress, apoptosis/necrosis, DNA adduct formation and cell cycle arrest were possibly involved in OTA toxicity. OTA intake increased some marker of liver damage such as AST, ALT, GGT and ALP [27], which may be caused by OTA-induced oxidative damage [28] and apoptosis [29]. OTA was reported to enhance lipid peroxidation [30,31], however, its influence on other aspects of lipid metabolism remains largely unknown. In the present study, we found that OTA increased lipid deposition and TG accumulation in liver, which revealed its influence on hepatic lipid metabolism and its risk to induce NAFLD. These findings have improved our understanding of this fungal toxin.

PPARs are representative members of nuclear receptors. This large superfamily is capable of ligand binding, which modulates their activities to regulate gene expression [32]. It has been determined that fatty acids and their derivatives bind and activate PPAR proteins [33]. Therefore, PPARs are important regulators to maintain cellular metabolic homeostasis. Lim et al. reported that OTA notably reduced the expression of adipocyte-specific genes, including PPARγ, therefore inhibited adipogenesis in mesenchymal stem cells derived from human adipose tissue [34]. In contrast, we found that PPARγ protein expression was increased in the livers after OTA treatment, whereas the mRNA level was comparable with control livers. This inconsistence may be attributed to the different cell types. It was reported that prolonged OTA exposure decreased ubiquitination levels of proteins by promoting proteasome activity [35]. However, we observed that OTA increased the PPARγ protein level in our study. We found that the interaction between PPARγ and its E3 ligase SIAH2 was reduced upon OTA treatment. Consequently, OTA prevented degradation of PPARγ. Therefore, OTA may influence protein stability in different ways.

Consistent with the increased expression and activity of PPARγ upon OTA treatment, the expression of CD36, a target of PPARγ [36] was increased in vivo and in vitro upon OTA treatment. CD36 is an important mediator of lipid uptake in many tissues, and abnormal CD36 expression in the liver resulted in TG accumulation and the development of hepatic steatosis [37]. As expected, OTA-induced lipid droplets formation and TG accumulation was alleviated in CD36 knockdown cells. FABP2 is involved in fatty acid transportation [38], and is another downstream target of PPARγ. Similar to CD36, expression of FABP2 was also increased after OTA treatment. Knockdown of *FABP2* reduced lipid droplets accumulation, but had no effect on TG contents. We noticed that OTA also upregulated the expression of two other FABPs, FABP1 and FABP3 (Figure 4d), although the alteration was less significant than FABP2. These FABPs may compensate for knockdown of FABP2, which contributed to the modest effect on lipid metabolism caused by FABP2 silencing. Therefore, CD36 seems the predominant effector downstream of PPARγ to mediate the effect of OTA on hepatic lipid metabolism.

In summary, the current study demonstrated that long-term exposure to OTA induces lipid accumulation in the liver of mice, mainly through activation of PPARγ signaling via post-translational modification of this nuclear receptor. Our study not only reveals the novel hepatic toxicity of OTA other than ROS generation and apoptosis induction, but also highlights the risk of OTA to cause NAFLD.

## 4. Materials and Methods

### 4.1. Experimental Animals

Six weeks old male C57BL/6 mice were purchased from LINGCHANG BIOTECH and were distributed to 2 groups with 12 each at random. The OTA group treated with 1 mg/kg. bw OTA (Aladdin, Shanghai, China) in 0.1 M NaHCO_3_ (100 µL) by gavage every day for 12 weeks. The control group treated with 0.1 M NaHCO_3_ similarly. Body weights were recorded weekly.

### 4.2. Cell Lines, Plasmids and Reagents

HepG2 cell line and pcDNA.3.1-Flag- PPARg2 plasmid were kindly gifted from Yu Li (SINH, CAS). HepG2, 293T cells and mouse primary hepatocytes were cultured in complete DMEM containing 10% FBS and 1% penicillin-streptomycin. shRNAs, targeted CD36 and FABP2, were inserted into pLKO. 1-TRC vector. The sequences of shRNA were listed in Table S1. GW9662 (HY-16578) and sulfosuccinimidyl oleate (HY-112847) were purchased from Medchemexpress (Shanghai, China) for in vitro studies.

### 4.3. Primary Hepatocytes Separation

Mouse primary hepatocytes were isolated from 8–12 weeks old mice by liver perfusion. Briefly, mice were anesthetized with 6% chloral hydrate. Buffer I (1 × EBSS and 0.5 mM EGTA) and Buffer II (1 × EBSS, 0.2 mg/mL collagenase IV, 10 mM HEPES and 2 mM CaCl) were successively perfused through the mice portal vein for 15 min. The liver was then removed, minced and strained through 70 µ um cell strainers. Single-cell suspensions were centrifuged at 800 rpm/min for 3 min, and hepatocytes were purified on 45% percoll.

### 4.4. Real-Time PCR

Total RNA from cells and tissues were extracted according to the protocol of our laboratory [39]. Real-time PCR was performed in triplicate using SYBR Green PCR Master Mix (Yeasen Biotech, Shanghai, China) on the ABI QuantStudio 6 PCR System (Thermo Fisher, MA, USA). Δct = ct (target gene) − ct(GAPDH), ΔΔct = Δct (experiment-control) and fold changes = 2^−ΔΔct^ 2^(−ΔΔct). Gene-specific primer pairs used in this study are listed in Table S2.

### 4.5. Western Blot Analysis

Western blot was performed as described previously [39]. Antibodies to PPARγ (C26H12) (2435S) and HA (C29F4) (3724S) were purchased from Cell Signaling Technology (Danvers, Massachusetts, USA); antibodies to SIAH2, CD36 and GAPDH were purchased from Proteintech (Wuhan, China); antibody to FABP2 was purchased from ABclonal (Wuhan, China); antibody to Lamin B1 was purchased from Abways Technology (Shanghai, China); and antibody to FLAG was purchased from Sigma-Aldrich (Saint Louis, MO, USA).

### 4.6. Oil Red O Staining

Liver tissues were imbedded into Tissue-Tek OCT compound and frozen for further experiments. Oil red O solution was mixed with distilled water at a ratio of 3:2, then the mixture was filtered by 0.4 μm filter. Frozen sections of liver (8 mm thick) were fixed in 10% buffered formalin for 15 min, rinsed with 60% isopropanol, then stained with Oil Red O for 15 min. After washing with 60% isopropanol, hematoxylin was used to stain the nuclei. Finally, covered the stained sections and microscopically examined.

### 4.7. Mouse Serum and Hepatic Lipid Analyses

Mouse serum was collected from heart blood with centrifugation at 6000 rpm for 25 min at 4 °C. For liver lipid tests, 50 mg liver tissue was homogenized in PBS, then lipids were extracted using a solvent mix containing methanol and chloroform (1:2). Total TG, ALT and AST in serum were measured using commercial kits (Shanghai Shensuo) according to the manufacturer’s protocol.

### 4.8. BODIPY Staining

Cells were fixed in 4% formaldehyde for 15 min on microscope slides, followed washing with PBS. Then staining with prepared BODIPY solution for 30 min at 37 °C. After washing, cells were re-stained with the hocheast for 3 min. Finally, the fluorescent data were obtained by confocal microscope (Zeiss, LSM 510 NLO).

### 4.9. RNA Sequencing and Processing

Total RNA was isolated from liver tissues of OTA and control mice. A total amount of 1 µg RNA per sample was used as input material for the RNA sample preparations. Sequencing libraries were generated using NEBNext^®^ UltraTM RNA Library Prep Kit for Illumina^®^ (NEB, USA) following manufacturer’s recommendations and index codes were added to attribute sequences to each sample. Differential expression analysis of two groups was performed using the DESeq2 R package (1.16.1). The resulting *p*-values were adjusted using the Benjamini and Hochberg’s approach for controlling the false discovery rate. Genes with an adjusted *p*-value < 0.05 found by DESeq2 were assigned as differentially expressed. Statistical enrichment of differential expression genes in KEGG pathways were tested using clusterProfiler R package. The whole procedure was performed by Novogene Co., Ltd. (Beijing, China).

### 4.10. Statistical Analysis

All data were conducted by GraphPad Prism 5.0 (Macintosh, San Diego, CA, USA). Data are presented as the mean ± S.E.M. Statistical differences between two experimental groups were determined by Student’s *t*-test. A *p* value < 0.05 was considered statistically significant.

## Data Availability

The data presented in this study are available on request from the corresponding author (D.X. and J.-J.L.), upon reasonable request.

## Supplementary Materials

The following are available online at https://www.mdpi.com/article/10.3390/toxins13110802/s1, Figure S1: OTA affects PPARγ signaling, Figure S2: FABP2 is involved in the effect of OTA on lipid metabolism in liver cells, Table S1: Primers of shRNA, Table S2: Primers for Real-Time PCR detection.

## Abbreviations

ALT, alanine transaminase; AST, aspartate aminotransferase; CHX, cycloheximide; NAFLD, nonalcoholic fatty liver disease; OTA, Ochratoxin A; SSO, sulfosuccinimidyl oleate; TG, triglycerides.
