# Supplementary material for: Ochratoxin A Induces Steatosis via PPARγ-CD36 Axis
Source: Toxins (Basel). 2021 Nov 13;13(11):802. doi: 10.3390/toxins13110802 (PMC8620754; doi:10.3390/toxins13110802)
Supplement: Supplementary file 1 [file toxins-13-00802-s001.zip › toxins-1440354-SI.pdf]

Supplementary Materials: Ochratoxin A Induces Steatosis via PPARγ-CD36 Axis

Qian-Wen Zheng, Xu-Fen Ding, Hui-Jun Cao, Qian-Zhi Ni, Bing Zhu, Ning Ma, Feng-Kun Zhang, Yi-Kang Wang, Sheng Xu, Tian-Wei Chen, Ji Xia, Xiao-Song Qiu, Dian-Zhen Yu, Dong Xie and Jing-Jing Li

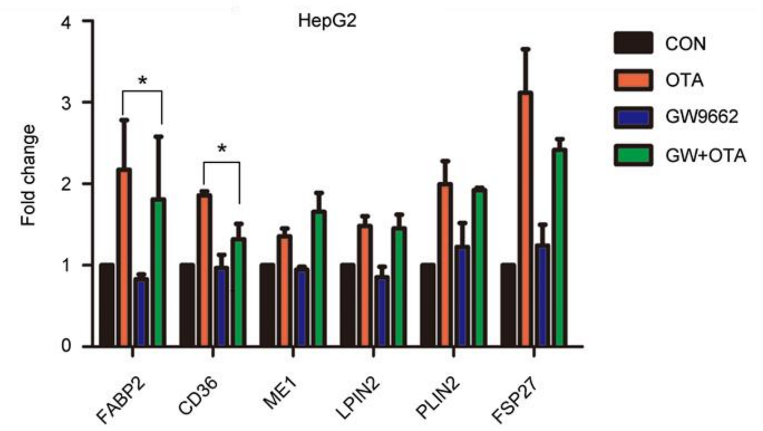

Figure S1. OTA affects PPARγ signaling. Fold change of gene mRNA expression by qPCR in HepG2 cells under indicated treatment (n = 6 biological replicates). Data shown as the mean ± S.E.M. * $P < 0.05$.

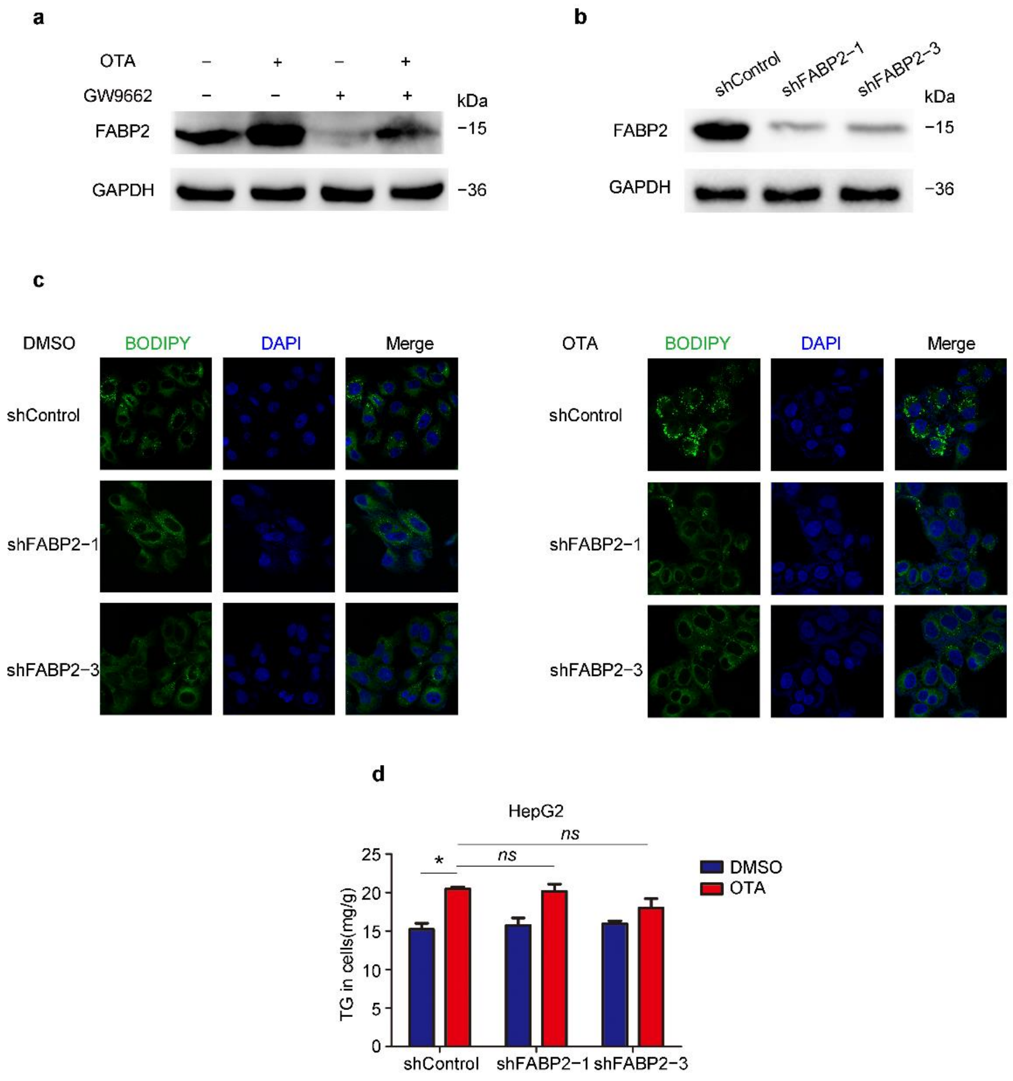

Figure S2. FABP2 is involved in the effect of OTA on lipid metabolism in liver cells. (**A**) Western blot analysis of FABP2 expression in HepG2 cells under indicated treatment. (**B**) Knockdown efficiency of FABP2 in HepG2 cells. (**C**) BODIPY staining of lipid droplets in control and FABP2-knockdown HepG2 cells treated with DMSO and OTA. (**D**) TG contents in control and FABP2-knockdown HepG2 cells treated with DMSO and OTA ($n = 6$ biological replicates). Data shown as the mean ± S.E.M. * $P < 0.05$, *ns* means no significant difference.

Table S1. Primers of shRNA.

Primers	Sequence 5'→3'
CD36 shRNA2	F: CCGGGCCATAATCGACACATATAAACTCGAGTTTATATGTGTCGATTATGGCTTTTTG
	R: AATTCAAAAAGCCATAATCGACACATATAAACTCGAGTTTATATGTGTCGATTATGGC
CD36 shRNA3	F: CCGGACGGCTGCAGGTCAACCTATTCTCGAGAATAGGTTGACCTGCAGCCGTTTTTTG
	R: AATTCAAAAAACGGCTGCAGGTCAACCTATTCTCGAGAATAGGTTGACCTGCAGCCGT
FABP2 shRNA1	F: CCGGTGGAGCCTTGAGGGAAATAAACTCGAGTTTATTTCCCTCAAGGCTCCATTTTTG
	R: AATTCAAAAATGGAGCCTTGAGGGAAATAAACTCGAGTTTATTTCCCTCAAGGCTCCA
FABP2 shRNA3	F: CCGGCGAGAAATTATAGGTGATGAACTCGAGTTCATCACCTATAATTTCTCGTTTTTG
	R: AATTCAAAAACGAGAAATTATAGGTGATGAACTCGAGTTCATCACCTATAATTTCTCG

Table S2. Primers for Real-Time PCR detection.

Gene	GenBank Accession Number	Sequence 5'→3'
Mouse Fabp2	14079	F: GCTGATTGCTGTCCGAGAGGTT
		R: AGCCTGGCATTAGCATGATGGA
Mouse Fads2	56473	F: GATGGCTGCAACATGACTATGG
		R: GCTGAGGCACCCTTTAAGTGG
Mouse PPARg	19016	F: GGAAGACCACTCGCATTCCTT
		R: GTAATCAGCAACCATTGGGTCA
Mouse Me1	17436	F: GCCGGCTCTATCCTCCTTTG
		R: TTTGTATGCATCTTGCACAATCTTT
Mouse Lpin2	64898	F: CAGAGTTCAGACGTTTCTCACAC
		R: GCTCCTTGTAGTCCTCTTCCT
Mouse Plin2	11520	F: CTTGTGTCCTCCGCTTATGTC
		R: GCAGAGGTCACGGTCTTCAC
Mouse Cd36	12491	F: ATGGGCTGTGATCGGAACTG
		R: GTCTTCCCAATAAGCATGTCTCC
Mouse Fsp27	14311	F: ATGGACTACGCCATGAAGTCT
		R: CGGTGCTAACACGACAGGG
Mouse Siah2	20439	F: CCAATGCCGCCAGAAGTTAAG
		R: CAGGGAAACAGAACTGCCGA
Mouse Gapdh	14433	F: AGGTCGGTGTGAACGGATTTG
		R: TGTAGACCATGTAGTTGAGGTCA
Human FABP2	2169	F: ATGGCGTTTGACAGCACTTG
		R: TCAGTTCCGTCTGCTAGATTGTA
Human FADS2	9415	F: GACCACGGCAAGAACTCAAAG
		R: GAGGGTAGGAATCCAGCCATT
Human LPIN2	9663	F: TCTACAAGGGCATTAACCAGGC
		R: AACGTGAAAAGGTGAACACTGA
Human PLIN2	123	F: TTGCAGTTGCCAATACCTATGC
		R: CCAGTCACAGTAGTCGTCACA
Human PPARG	5468	F: GGGATCAGCTCCGTGGATCT
		R: TGCACTTTGGTACTCTTGAAGTT
Human FSP27	63924	F: AAGTCCCTTAGCCTTCTCTACC
		R: CCTTCCTCACGCTTCGATCC
Human GAPDH	2597	F: CTGGGCTACACTGAGCACC
		R: AAGTGGTCGTTGAGGGCAATG
Mouse/Human MKRN1	Human: 23608	F: GAGCAGGTTCAGAGGACTGG
	Mouse: 54484	R: CACTCTCCCACTGCAGCATA
Human SIAH2	6478	F: CGCCAGAAGTTGAGCTGCT
		R: TGGTGGCATACTTACAGGGAA
